# Rethinking CSR theory to incorporate microbial metabolic diversity and foraging traits

**DOI:** 10.1038/s41396-023-01486-x

**Published:** 2023-08-18

**Authors:** J. L. Wood, A. A. Malik, C. Greening, P. T. Green, M. McGeoch, A. E. Franks

**Affiliations:** 1 Department of Microbiology, Anatomy, Physiology and Pharmacology, La Trobe University, Melbourne, VIC Australia; 2 Research Centre for Future Landscapes, La Trobe University, Melbourne, VIC Australia; 3 School of Biological Sciences, University of Aberdeen, Aberdeen, AB24 3UU UK; 4 Department of Microbiology, Biomedicine Discovery Institute, Monash University, Clayton, VIC 3800 Australia; 5 Securing Antarctica’s Environmental Future, Monash University, Clayton, VIC 3800 Australia; 6 Department of Environment and Genetics, La Trobe University, Melbourne, VIC Australia

**Keywords:** Community ecology, Microbial ecology, Microbial ecology, Theoretical ecology

Microbial communities support the health and function of individual organisms, all the way up to global biomes. Given rapidly changing global ecosystems, there is an urgent need to be able to predict what changes to microbial community structure mean for the emergent community function. The use of microbial functional traits presents an opportunity for describing microbial communities in terms of ecologically relevant and meaningful processes that can be embedded into quantitative and theoretical frameworks.

Functional traits, hereafter referred to as traits, may be any morphological or physiological characteristic that determines fitness in a given environment [1, 2]. The genetic proxies for these expressed characteristics can also be treated as traits. For example, bacterial motility machinery is encoded on a known suite of genes, and rRNA copy number is thought to broadly correlate with growth rate. Traits are commonly regarded at either a species or individual organism level. However, the abundances of all species in a community that express a certain trait can be amalgamated to determine community-aggregated traits. In microbial ecology, functional trait analyses frequently aim to find characteristics of the collective microbial community (i.e., community-aggregated traits), which can be compared across different treatments or environments, and used to investigate the function of ecosystems. Because environmental selection acts on traits, trait-based composition-environment relationships can theoretically be identified even among communities that have few or no species in common. This feature allows trait analyses to facilitate the identification of community-level patterns among disparate microbial communities, with the potential even to compare communities from different trophic levels [1, 3]. Moreover, because communities accumulate traits in response to their environment over time, the accumulated genomic traits of a community could help infer its evolutionary history and adaptations.

While trait-based approaches can be used to identify general patterns and trends in microbial strategies, trait analyses alone are not predictive. To predict how microbiome function will be altered by a change in the environment, trait analyses need to be incorporated into ecological frameworks that enable general patterns to be explored, tested and established. One such framework is Grime’s Competitor, Stress-tolerator, Ruderal (CSR) theory (see Box 1) [4]. The CSR framework was originally developed for plant communities and has been applied to microorganisms, to predict specific relationships [5] and to understand key ecosystem functions such as carbon cycling [6] and phytoremediation [7]. The practical application and potential management benefits arising from the application of CSR frameworks to microbial communities are clear. However, a different application of trait frameworks is to facilitate the formation of universal ecological hypotheses by using them to compare disparate communities. Such hypotheses represent a major advance to the field of microbial ecology and, potentially, to ecology more broadly through a taxonomically inclusive theoretical framework for life strategies. Of the trait measures available to achieve this task, genetically encoded community-aggregated traits hold much promise. Unlike expressed traits, which can provide insight into a community’s activities under a given set of conditions, genetically encoded traits reveal both the cumulative impact of selection on a given community as well as the community’s latent capacity to respond to future change. In this way, genetically encoded traits provide a path toward improving predictive capacity.

Here we outline the development of an ecological framework for comparing the life history strategies of microbial communities across distinct biomes. We begin by examining the definitions underpinning Grime’s CSR framework, highlighting the potential and current limits in applying CSR theory to microbiota. We examine the various interpretations of CSR definitions in microbial ecology to identify key microbial traits whose CSR classifications lack consistency. Then, using these traits, we propose a new set of CSR definitions resulting in a new framework, the CSO framework, within which all microbial life strategies are accommodated. We revisit microbial traits identified as having inconsistent CSR classifications and discuss how they fit within the new CSO framework. Finally, we outline how our framework can be applied to study microbial communities and the next steps toward a practical application of CSO.

## Applying CSR theory to microbial communities

Grime’s CSR theory was first proposed in the 1970s, and it remains a valuable framework that links environmental condition to community composition and species attributes (see Box 1). Microbial community ecologists are particularly well positioned to develop CSR theory further because microbial community-level CSR scores can be captured in a high throughput manner directly from trait measures using the abundance of encoded gene content. This contrasts with plant-community CSR scores, which rely on calculating and then agglomerating species-level CSR scores into a community score. The capacity for high-throughput analysis coupled with direct gene-level trait measurements makes it possible to build on, develop and test CSR-like frameworks without making assumptions regarding intra-species trait variation.

The current marrying of microbial community data with CSR definitions is promising but by no means perfect and requires further conceptual development [6]. The first conceptual difficulty lies in the fact that CSR theory was designed to describe resource-gathering and -allocation strategies in photosynthetic organisms. However, microorganisms are notoriously metabolically flexible, able to gain their energy from light, organic compounds, inorganic matter, and even thin air. This is problematic for researchers seeking to compare connected and interacting communities that occur across an environmental boundary. For example, top and sub-soil communities share a physical connection and interact through nutrient cycling but may be dominated by aerobic and anaerobic metabolisms respectively. How can we compare the ecology of these communities beyond quantifying their differences in metabolism and energy requirements? To meaningfully compare communities with differing metabolic diversity and/or dominant metabolic processes, the trait-based classification frameworks must be general enough to encompass all modes of metabolism. This challenge has not been resolved to date; however, numerous attempts to apply CSR theory to metabolic subsets of microbial communities have been made [5–9]. In addition, traits must be responsive to environmental change and have a meaningful connection to the collective fitness of a community.

Looking across existing microbial CSR conceptualisations, some microbial traits appear to fulfil these prerequisites and also fit neatly within current CSR definitions (Table 1). For example, ‘antibiotic production’ is universally predicted to be a competitive trait [5, 7, 8]. This fits well with the CSR definition of a competitive trait, which is ‘One that facilitates resource monopolisation via the inhibition of resource capture in neighbours’ and is supported by experimental evidence from microcosm [10] and cross-biome studies [11] of soil communities. Similarly, it is generally accepted that organisms designated as ruderal (R) life strategists are likely to exhibit some measure of rapid growth (e.g., responsiveness, high respiration rate, high rRNA operon copies). This classification makes intuitive sense and draws on parallels with the rapid growth and life cycles of ruderal or ‘weedy’ plants.

**Table 1:** Summary of CSR trait definitions as outlined in Grime and Pierce [12] and adaptions to these definitions, proposed in this perspective, to incorporate microbial communities.

Trait classification	Current (CSR) definition	Proposed (CSO) definition
Competitor (C) trait	Traits constituting an investment in monopolising local resources in potentially productive environments	Traits that facilitate monopolisation of resources in environments where resource-use is unconstrained
Stress-tolerator (S) trait	Traits that facilitate survival in chronically underproductive environments.	Traits that facilitate survival in environments where resource-use is constrained
Ruderal (R) trait/opportunist (O) trait	Traits constituting an investment in processes that permit the re-establishment of a population	Traits that facilitate the monopolisation of resources in environments where resource-use constraint is dynamic or temporally variable

Microbial foraging traits, such as chemotaxis and motility, have a more problematic relationship with Grimesian definitions. Foraging traits are most typically predicted to be traits of R life strategists [5–7], but have also been defined as competitive [7]. The rationale for classifying foraging traits as ruderal draws on parallels between macro and micro, likening the high rates of dispersal and colonisation seen in ruderal plants to microbial foraging [5]. With the aim of integrating CSR theory into microbial ecology, Krause et al. (2013) classified foraging traits as ruderal by adjusting the definition of a ruderal trait to one that ‘facilitates the exploration and exploitation of an environment’. Indeed, in a worked example extending CSR Theory beyond plants, Grime and Pierce [12] draw similar parallels by predicting the traits of an R-selected mammal would encompass those that facilitate being ‘opportunistic in ephemeral niches’. While drawing parallels between plant and microbial foraging/dispersal strategies makes intuitive sense, the CSR definition of a ruderal trait is one that constitutes an investment in processes that ‘permit the re-establishment of a population after a disturbance’ (Table 1). Disturbance is defined by Grime as ‘the partial or complete destruction of biological material’ [12]. The role of disturbance in ruderal life strategies is not captured in Krause’s redefining of a ruderal trait, making it difficult to overlay microbial CSR-allocated traits on traditional CSR axes of ‘increasing disturbance’ and ‘increasing stress’ (Box 1). Bacterial foraging traits tend to prevail in environments where resources show spatial and temporal variability in their availability, such as the photic zone of the ocean water column [13]. While these communities may experience disturbed or interrupted resource supply, they do not necessarily experience disturbance as defined by Grime (Table 1).

One set of microbial traits that do respond to disturbance is the ability to form spores or other resting structures such as viable nonculturable states. Following a disturbance where a portion of the community has been destroyed, such as wildfires or sterilisation, spore-forming microorganisms proliferate and dominate [14]. Yet, despite being one of the few traits clearly linked to disturbance, microbial conceptualisations of CSR theory largely allocate spore formation as a trait belonging to S-selected organisms owing to the ability of spores to aid persistence in an underproductive environment [5, 7]. It may be that ecological theories developed for macro-organisms cannot be appropriately translated to microbial communities. However, it is equally possible that ecological theories formed in the absence of knowledge of microbial communities are simply incomplete. Rethinking CSR theory with respect to bacterial foraging traits lends weight to the latter possibility.

Box 1 Diagrammatic representation of the three-way trait trade-off for plants **a** as conceptualised by Grime and **b** projected onto the axes ‘stress’ and ‘disturbance’ highlighting the absence of life strategies pertaining to a fourth set of conditions: high disturbance and high stress. Grime’s CSR life strategy theory predicts that organisms face a three-way resource trade-off between the investment in competitive (C), stress-tolerant (S) or ruderal (R) traits that is governed by levels of stress and disturbance present in an environment. The theory predicts that in scenarios of low stress + low disturbance organisms that exhibit competitive life strategies will prevail. In this scenario, the investment of resources into competitive traits, such as large root systems, canopies or allelochemical production, confers a selective advantage that outweighs the loss in fitness due to reduced investment in other adaptive strategies, such as stress tolerance or colonisation potential. In high stress + low disturbance scenarios, the theory predicts that stress-tolerant life strategists will prevail. These tend to be slow growing investing in traits that facilitate maintenance or biomass retention (e.g., detoxification mechanisms and mechanical or chemical defences). Conversely, the theory predicts in low stress + high disturbance scenarios, ruderal traits, which pertain to re-colonisation potential (e.g., short life cycle, high seed count, dispersiveness) will confer a selective advantage and ruderal life strategists will prevail. The theory does not venture predictions for a high stress + high disturbance scenario.
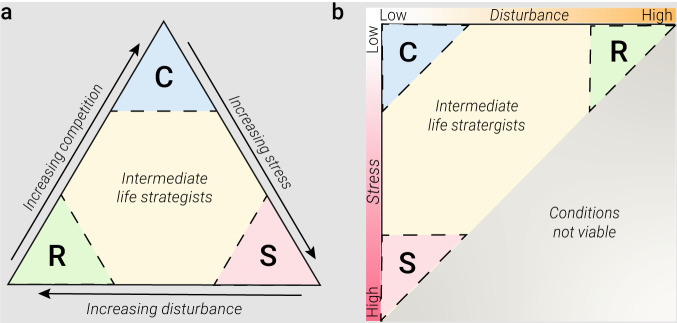

## A ‘micro’ revision of Grime’s CSR framework

Foraging traits can be incorporated into a three-way trade-off scheme by reconceptualising the CSR axis to reflect the way microbial communities experience resource constraints in the landscape. The traditional C–S axis is one of increasing stress, where stress may be any intrinsic or extrinsic factor that constrains growth. Stress can include physico-chemical limits (i.e., extremes of temperature, pH, water availability) and the presence of pollutants, all of which require resources to be diverted from growth and invested instead in cell survival or detoxification strategies. Environments where carbon is inaccessible or of low quality can also be thought of as high on the stress axis. In these environments growth is constrained because resources are diverted to produce enzymes necessary for mobilising or degrading complex carbon substrates. We might think of the C–S axis as one of increasing resource-use constraint, where resources are increasingly diverted from growth into activities that assist the organism with managing environmental constraints. In conditions of low resource-use constraint, resources will be invested into growth and monopolising the local carbon pool (vis. competitive life strategies). The definition of C-selected traits changes only marginally under this re-working, with the selective environment redefined from one that is ‘potentially productive’ to one where ‘resource-use is unconstrained’ (Table 1). Similarly, the definition of an environment that selects for stress tolerance traits needs to be updated from one that is ‘chronically underproductive’ to one which is ‘resource-use constrained’.

**Fig. 1:**
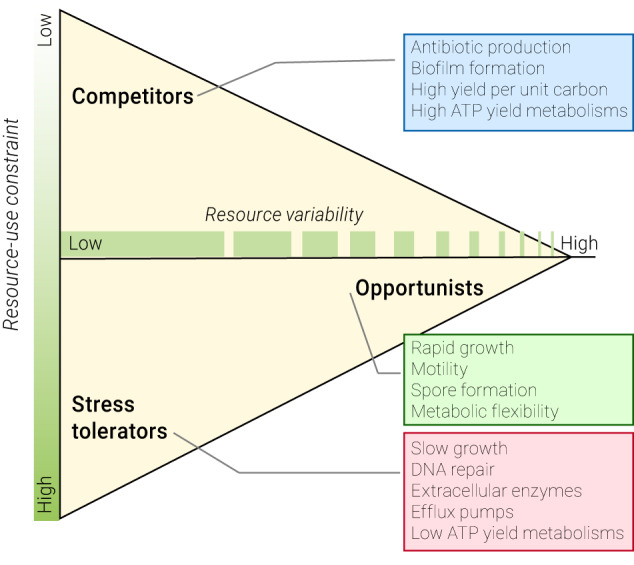
Diagram of the CSO three-way life strategy trade-off as interpreted in terms of resource-use constraint and resource variability to incorporate microbial foraging strategies and metabolic diversity. Microbial traits hypothesised to typify each life strategy are highlighted in call-out boxes.

In addition to intrinsic and extrinsic stressors, resource-use can also be constrained by the type of electron donor-acceptor pairings available in the environment. Aerobic respiration using organic carbon is the most efficient mode of metabolism, typically yielding some 38 ATP molecules, imposing the least constraint on growth. In contrast, fermentation is considerably more constrained typically yielding only 2 ATP, while the many and various forms of anaerobic respiration produce somewhere between these two extremes. Despite CO_2_ and sunlight being freely available in many environments, oxygenic photosynthetic organisms also experience a level of resource-use constraint. This is because a by-product of photorespiration is the accumulation of reactive oxygen species in the cell and resources must be diverted to detoxify them [15]. By considering the C–S axis one of resource-use constraint (Fig. 1), traditional CSR is expanded to incorporate the multiple metabolic strategies that microorganisms can use.

Microbial foraging traits can now be incorporated into CSR theory by reconceptualising the R axis, from one of increasing disturbance, to one of increasing resource variability, which can also be thought of as the frequency with which an environment transitions between states of high and low resource-use constraint. Redefining a ruderal trait as one that ‘facilitates the monopolisation of resources in environments where resource-use constraint is dynamic or temporally variable’ expands the notion of ruderal traits from those that permit primary colonisation following biomass destruction to include traits that facilitate primary colonisation of resources in resource-patchy environments. We have re-named the latter to ‘opportunists’ or O strategy to encompass the notion of a foraging lifestyle (Fig. 1). As with the concept of resource-use constraint, there are multiple factors that can contribute to resource variability in the landscape, including spatial and temporal factors. Spatial variability may arise in environments where resources are always present but discontiguous or ephemeral in nature, for example, the presence of falling organic particles (marine snow) in the upper ocean. Temporal variability arises due to release from resource-use constraints imposed by the environment and may occur over frequent (e.g., repeated inundation of intertidal environments) or infrequent time periods (e.g., rain events in a desert).

Under our revised conceptualisation, the Competitor, Stress-tolerator, Opportunist (CSO) framework, the axes along which traits are distributed move from being ‘stress’ and ‘disturbance’, both biocentric concepts relating to the impediment of biomass formation and to biomass destruction, respectively, to being ones that are resource-centric: resource-use constraint and resource variability (Fig. 1). The *x*-axis separates communities with life history strategists adapted to transient resource availability from those that are not, while the *y*-axis separates communities with life strategies for managing biological resource competition from those managing environmentally imposed resource-use constraints. The new definitions, summarised alongside CSR definitions (Table 1), reconcile CSR trait allocations for microbial communities whilst retaining their original meaning for plant communities.

## Reconciling microbial traits within the new CSO framework

These revised definitions and resource-centric axes resolve the allocation of survival strategies including spore formation and dormant states as being O selected. Previously, spore formation was generally hypothesised to be aligned with S-selection as it fit with the Grimesian definition of ‘traits facilitating survival…’. We argue that traits are only truly S-selected if the organism is still actively growing and contributing to the wider ecosystem. As these traits predominantly assist growth following periods of release from resource-use constraint, their selection is driven by resource variability, specifically cases where variability occurs between long periods of stability (Fig. 1).

The CSO framework also reconciles two wider issues with CSR as it applies to microorganisms. First, the issue of a three-way trade-off system for microbial ecologists. Grime’s CSR environmental classifications of low stress + low disturbance, low stress + high disturbance, and high stress + low disturbance are notably missing the environmental combination of high stress + high disturbance (Box 1). The rationale for this exclusion is that plants cannot survive and reproduce under such conditions [12]. However, this rationale does not hold for microorganisms, which are able to inhabit a wide variety of extreme environments [6]. The revised CSO definitions reduce the environmental conditions to three: high resource stability + low resource-use constraint (C-selected), high resource stability + high resource-use constraint (S-selected), and low resource stability (O selected). As discussed, ‘resource variability’ includes cycles of resource-use constraints and release from those constraints; thus, all environmental conditions are accounted for.

As already discussed, a second major challenge involves creating a framework that encompasses the great metabolic diversity present among microorganisms. Because microorganisms can gain their energy from diverse sources, the question of ‘What resource are we building the framework on?’ becomes very pertinent. The question can be resolved if we consider the two major roles of resources: (1) resources needed to make new biomass and (2) resources needed to transduce the energy to create that biomass. The former is unequivocally carbon—the building block of life—while the latter can be thought of as a strategy for acquiring the former. It is true that any nutrient can limit biomass production, but our focus on carbon as the key biomass resource allows for generalisation. Strategies to acquire other limiting nutrients (e.g., Fe via siderophores, P via P-solubilisation, N via N-fixation) are not ubiquitous and therefore do not fulfil our criteria of ‘being independent of or encompassing all metabolic strategies’. Such traits may well be valuable as secondary or explanatory traits for further investigating a community’s ecology beyond its broader ecological classification. Similarly, to classify S-selected communities, our framework focuses on general stress traits, such as slow growth rate and investment in repair machinery. As with specific nutrient acquisition strategies, we expect traits related to specific resource constraints (e.g., heavy metal resistance genes in the presence of heavy metals) to fulfil the role of secondary or explanatory traits.

Using the above perspective, the resource upon which the CSO definitions are built is firmly set to be carbon while energy metabolism becomes a trait that can be associated with existing life history strategies. Energy metabolism is already partially incorporated into the CSO framework by considering the availability of different electron donor-acceptor pairings as a gradient of environmental constraint. We can further relate energy metabolism to the O-axis by considering the breadth of energy metabolisms prevalent within a community. This gives rise to a general energy metabolism trait—metabolic diversity—that can be applied across all communities and biomes. Intuitively one might expect metabolic diversity to increase as environmental variability increases; thus, we predict it to be an O selected trait. Indeed, such a prediction has already been observed at the species level, where the frequently disrupted ‘mixing’ layer of sandy sediments was dominated by bacteria with higher metabolic flexibility compared to the more stable, sub-surface sediment layers [16].

In practice, few communities will be C, S or O selected in an absolute sense, but likely to represent some balance among the three, with intermediate classifications such as CO or SO likely. When using genetically encoded traits, these intermediate classifications can reveal how the community experiences an environment in a longer time frame. For example, while communities constantly in a state of flux may be O selected, more often instability is interspersed with periods of stability, whether these periods of stability are ones of resource abundance, or resource-use constraint will determine whether communities are characterised as CO or SO.

## Translatability and concluding remarks

Trait-based research can identify meaningful ecological trends across disparate microbial communities. The development of trait-based ecological frameworks, such as the CSR theory, will be paramount in explaining community-level changes in an ecologically meaningful way. It will also be integral for making predictions about how environmental and anthropogenic changes affect microbial communities. Indeed, existing microbial trait theories based on expressed traits are already being implemented in the literature to understand key community functions in terrestrial environments [17]. Our proposed amendments to CSR definitions reveal a new framework, the ‘CSO framework’, that is valid across disparate microbiological disciplines and resolves previously identified limitations of CSR’s applicability to microorganisms.

While the new axes are resource-centric, our focus on traits that are general across metabolic strategies allows the notion of ‘environment’ to be scale-flexible. That is to say, ecological strategies could be compared within one environment type or across environmental boundaries. This scalability opens multiple applications for the CSO framework. For example, an understanding of the CSO makeup for a well-functioning community (i.e., healthy gut, productive soil) can be used as a baseline to determine how a poorly functioning community of the same environment has diverged in its ecology. Because CSO traits describe how a community is being shaped by an environment, knowledge of how ecologies are diverging can reveal management actions for shifting a community from one state to another. Conversely, understanding the CSO makeup of communities that sit across an environmental boundary (e.g., top and sub-surface soils, oxic and anoxic zones, the small and large intestine) could be used to understand the functional intersection of the two environments, as well as trace and track the movement of transition zones between them.

Future research using controlled microcosms with defined gradients of resource-use constraint and resource variability will be needed to confirm core predictor traits that discriminate between C, S and O life strategies. These traits need to be general across metabolisms, responsive to environmental change, measurable for any environment and have a meaningful connection to community fitness. Ideally, identified trait trade-offs will be validated at the organism level to unpack how shifts in community-level traits reflect physiological trade-offs. The adoption of the CSO framework will assist in synthesising findings from across the microbial literature and aid the development of broad ecological theories for microbial ecology and potentially ecology more generally.
